# Cardiovascular secondary prevention in primary care setting: an immediate necessity in Brazil and worldwide

**DOI:** 10.1590/1516-3180.2017.1355190817

**Published:** 2017-08-21

**Authors:** Paulo Andrade Lotufo

**Affiliations:** I MD, DrPH. Full Professor, Department of Internal Medicine, Faculdade de Medicina da Universidade de São Paulo (FMUSP), São Paulo (SP), Brazil.

Cardiovascular diseases (CVD) such as coronary heart disease, stroke and peripheral arterial diseases are the leading causes of death worldwide.^1^ The decline in age-adjusted mortality rates observed since the late 1960s was initially attributed partially to two concomitant factors. First, case-fatality rates have decreased due to better awareness of early symptoms and introduction of pre-hospital care, coronary care units, new drugs and surgical interventions. Second, reduction in the numbers of cigarettes smoked per day (smoking has been correlated with sudden death due to cardiac arrest) and lowering of very high blood pressures (hypertension has been correlated with massive hemorrhagic stroke) have played a significant role in reducing the risk of death due to cardiovascular diseases.^2^ However, in contrast to assessments on mortality data, finding information about the incidence of CVDs is not an easy task. There are few empirical population-based studies, and extrapolation from mortality data with the aim of calculating incidence is insufficient.

We make the assumption that reductions in new-onset CVD cases are not occurring worldwide, or at least at the same pace observed for death rates. This assumption can be explained in terms of lifestyle changes that occurred during the 1990s. Primordial prevention (smoking bans, greater availability of healthy foods and encouragement of leisure-time physical activity) and more adequate primary prevention such as control of high blood pressure and high cholesterol became a reality.^3^ However, a new factor rapidly spread worldwide: the epidemic of obesity and diabetes, which has increased the number of people prone to CVD events.^4^

If the rates of new-onset cardiovascular diseases do not decline, but the case-fatality rates drop, the prevalence of people with CVD should consequently increase. The confluence of these movements (declining lethality, stable incidence and rising obesity) has increased the numbers of survivors from myocardial infarction and stroke. These individuals, together with the pool of people with angina pectoris and peripheral artery disease, are responsible for the increase in cardiovascular disease prevalence rates. Data from the 2013 Brazilian National Health Survey and the Brazilian Longitudinal Study of Adult Health (ELSA-Brasil) revealed that higher prevalence of these conditions was strongly associated with lower socioeconomic status^5^^,^^6^^,^^7^ (Table 1). Consequently, the prevalence of individuals who can be assigned to secondary prevention is growing mainly among the poorest.

**Table 1:** Prevalence ratios (and 95% confidence intervals) according to level of education, for heart diseases, angina and stroke from the 2013 Brazilian National Health Survey (NHS) and for peripheral artery disease from the Brazilian Longitudinal Study of Adult Health (ELSA-Brasil)^5^^,^^6^^,^^7^

Education level	2013 NHS (n = 60,202)			ELSA-Brasil (n = 15,105)
	Heart disease	Angina pectoris	Stroke	Peripheral artery disease
Less than elementary	6.3 (5.7-6.9)	10.8 (10-11.5)	2.7 (2.4-3.1)	10.1 (9.5--0.6)
Elementary	3.1 (2.4-3.7)	7.5 (6.5-8.5)	0.8 (0.6-1.1)	8.8 (8.6-9.1)
High school	2.5 (2.0-2.9)	5.5 (4.9-6)	0.8 (0.8-1)	5.5 (5.4-5.7)
College	3.5 (2.5-4.4)	3.6 (2.9-14.4)	0.6 (0.6-0.8)	5.4 (5.3-5.5)

Although the benefits of secondary prevention measures (lifestyle changes, blood pressure control and use of aspirin and statins) to reduce health-adverse cardiovascular events is undisputable,^8^ these measures have not been adopted worldwide. In the Prospective Urban Rural Epidemiological (PURE) study, data on 153,996 adults with CVDs (aged 35-70 years) who were living in countries at different stages of economic development were analyzed. Comparing South America with North America/Europe, the rates of use of medications of proven effectiveness for people with coronary heart diseases (CHDs) or stroke were, respectively, 29% versus 52% for antiplatelet drugs; 58% versus 69% for blood pressure lowering agents; and 15% versus 52% for statins.^9^ One explanation for these results is that neither outpatient cardiological units, nor primary care facilities in South America have adopted trustworthy programs for secondary prevention as a public health policy.

One challenge is to test how secondary prevention as provided by several guidelines should be practiced within primary care settings.^8^ The problem of “secondary” (cardiovascular prevention) within the “primary” (care setting) is much more related to phraseology than to the capabilities and features of primary care units, including family health programs, which would undertake these measures immediately.
